# The Importance of Non-neuronal Cell Types in hiPSC-Based Disease Modeling and Drug Screening

**DOI:** 10.3389/fcell.2017.00117

**Published:** 2017-12-19

**Authors:** David M. Gonzalez, Jill Gregory, Kristen J. Brennand

**Affiliations:** ^1^Medical Scientist Training Program, Icahn School of Medicine at Mount Sinai, New York, NY, United States; ^2^Department of Developmental and Stem Cell Biology, Icahn School of Medicine at Mount Sinai, New York, NY, United States; ^3^Black Family Stem Cell Institute, Icahn School of Medicine at Mount Sinai, New York, NY, United States; ^4^Instructional Technology Group, Icahn School of Medicine at Mount Sinai, New York, NY, United States; ^5^Department of Neuroscience, Icahn School of Medicine at Mount Sinai, New York, NY, United States; ^6^Friedman Brain Institute, Icahn School of Medicine at Mount Sinai, New York, NY, United States; ^7^Department of Genetics and Genomics, Icahn School of Medicine at Mount Sinai, New York, NY, United States

**Keywords:** human induced pluripotent stem cells, drug screening, glia, schizophrenia, ALS, Rett syndrome

## Abstract

Current applications of human induced pluripotent stem cell (hiPSC) technologies in patient-specific models of neurodegenerative and neuropsychiatric disorders tend to focus on neuronal phenotypes. Here, we review recent efforts toward advancing hiPSCs toward non-neuronal cell types of the central nervous system (CNS) and highlight their potential use for the development of more complex *in vitro* models of neurodevelopment and disease. We present evidence from previous works in both rodents and humans of the importance of these cell types (oligodendrocytes, microglia, astrocytes) in neurological disease and highlight new hiPSC-based models that have sought to explore these relationships *in vitro*. Lastly, we summarize efforts toward conducting high-throughput screening experiments with hiPSCs and propose methods by which new screening platforms could be designed to better capture complex relationships between neural cell populations in health and disease.

## Introduction

The development of successful treatments for neurological disease is hampered by a constellation of unique challenges that has resulted in a historically poor rate of success in drug development in this area (Ringel et al., 2013). Many neurological diseases are complex and heterogeneous in nature, exhibiting a breadth of genetic and epigenetic variants of small effect sizes which incompatible and/or impractical to model using traditional *in vitro* and *in vivo* models (Sullivan et al., 2012; Fass et al., 2014). Furthermore, the use of rodent models may be insufficient to capture the complexity of human disease, as large differences in neurogenesis, neuroanatomy and distribution of neural cell types within the brain exist between mice and humans. Lastly, the lack of accessibility of neural tissue (both living and post-mortem) combined with difficulties in culturing these cell types *in vitro* has made developing cell culture models of neurological disease exceedingly difficult and slowed understanding of the pathophysiology underlying a number of these conditions. Human induced pluripotent stem cells (hiPSCs) now offer a nearly limitless potential for disease modeling and drug screening applications. Their great self-renewal and wide differentiation capacity, coupled with the relative ease of producing patient-specific hiPSCs harboring genetic variations implicated in disease, makes possible the generation of large quantities of diverse cell types in a controlled and iterative manner, ideal for high throughput screens to discover and evaluate the efficacy and safety of novel therapeutics (Haggarty and Perlis, 2014; Mertens et al., 2016). In recent years, stem cell models of complex genetic diseases have helped to shed new light on the pathology of various neurodegenerative and neuropsychiatric disorders (Di Giorgio et al., 2008; Dimos et al., 2008; Park et al., 2008; Soldner et al., 2009; Brennand et al., 2011; Yagi et al., 2011; Yoon et al., 2014).

Until recently, studies of mechanism and pathology of neuropsychiatric disorders have tended to focus predominantly on neurons, with little recognition of the complex milieu of cell types that interact with these cells and influence their function. Fortuitously, our newfound ability to generate a variety of cell types found in the central nervous system (CNS) from hiPSCs now presents an exciting opportunity to explore how these various cell types interact with one another in a controlled manner. In this review, we briefly overview current advances toward generating the major neural cell types—neurons, astrocytes, oligodendrocytes and microglia—using stem cell technologies. Further, we highlight recent advancements in understanding non-neuron cell-autonomous effects in the pathology of three representative neuropsychiatric disorders—Amyotrophic Lateral Sclerosis (ALS), schizophrenia and Rett Syndrome—both *in vitro* and *in vivo*, with an eye toward using this information to develop hiPSC-based drug screening platforms that better capture disease pathology.

## Progress in cellular reprogramming strategies to generate major CNS cell types

### Neurons

The lack of live patient neural tissue, combined with the limitations of post-mortem brain tissue, has spurred efforts to find new methods of models of CNS diseases. Stem cell based technologies, particularly the ability to reprogram induced pluripotent stem cells (hiPSCs), presents a powerful opportunity to create scalable, easily perturbed platforms to study how various genetic abnormalities implicated in disease manifest in altered neural cell function. There exist two general strategies for the creation of neurons using hiPSC-based technologies, directed differentiation and induction, each having advantages and disadvantages that Should be carefully considered when developing an appropriate platform for drug development.

Directed differentiation of iPSCs involves the sequential addition of growth factors and small molecules intended to recapitulate the embryonic developmental cues that drive the generation of mature tissue and different cell types *in vivo*. Neuronal differentiation protocols typically proceed via a neural progenitor cell (NPC) intermediate, a population of cells with the capacity to self-renew and to generate both neurons and glia. The application of a variety of mitogens can confer different positional identities and enrich for specific subtypes of neural cells. For a more in depth review of these differentiation methods and their developmental basis, an excellent review has been published elsewhere (Mertens et al., 2016). Conversely, neuronal induction proceeds via the forced overexpression of key neuronal transcription factors. When first reported, induced neurons were generated through the overexpression of three factors—*BRN2* (also known as POU3F2), achaetescute homolog 1 (*ASCL1*) and myelin transcriptional factor1-like protein (*MYT1L*)—in mouse or human fibroblasts (Vierbuchen et al., 2010; Pang et al., 2011; Son et al., 2011; Vadodaria et al., 2016). Collectively known as the BAM factors, they rapidly yielded a heterogeneous population of induced neurons that formed synapses and fired action potentials. A growing understanding of the key transcription factors responsible for assigning regional specificity and neurotransmitter identity has expanded our ability to induce specific populations of excitatory (*NGN2*) (Zhang et al., 2013), inhibitory (*ASCL1, LHX6, DHX2*, miR9/124) (Sun et al., 2016), dopaminergic (*MASH1, NURR1, LMX1A*) (Caiazzo et al., 2011) and serotonergic (*ASCL1, NGN2, NKX2.2, FEV, GATA2, LMX1B*) (Vadodaria et al., 2016; Xu et al., 2016) neurons for fibroblasts, hiPSCs and/or NPCs.

The choice between directed differentiation and neuronal induction approaches generally reflects needs of the experiment in question. From a practical standpoint, differentiation of iPSCs is costly and time consuming, often requiring 4–6 months to yield functional neurons. In contrast, while induced neurons can be generated from fibroblasts within 1–3 weeks (without the need to reprogram and validate hiPSCs), the populations produced tend to include large numbers of fibroblast-like contaminants and the absolute number of neurons that can be generated is less, reflecting the more limited replicative capacity of fibroblasts. A compromise of inducing neurons directly from hiPSCs promises a larger number of rapidly maturing neurons and may grow more common as subtype-specific induction methodologies become more refined.

Several fundamental differences distinguish neurons generated by directed differentiation or induction. First, chromatin remodeling is an essential part of the cellular reprogramming process; while important epigenetic markers associated with complex genetic diseases may be lost during hiPSC reprogramming, this does not seem to occur to the same extent during neuronal induction (Huh et al., 2016; Mertens et al., 2016). It is thought that cellular division during directed neuronal differentiation requires broad transcription factor access to previously closed regions, thus changing the epigenetic landscape of the cell in question. In contrast, neuronal induction does not require proliferation; the pioneer transcription factors seem to not require extensive chromatin remodeling to complete this process (Liu et al., 2013; Iwafuchi-Doi and Zaret, 2014; Fishman et al., 2015) and so may better preserve epigenetic signatures. Second, the establishment of an embryonic-like state during the reprogramming process is now understood to result in terminally differentiated phenotypes that resemble fetal rather than adult cells (Mariani et al., 2012; Nicholas et al., 2013; Brennand et al., 2015), suggesting that hiPSC-based models may better capture the genetic elements of disease predisposition rather than the disease-state itself. Although novel strategies to accelerate aging *in vitro* are being uncovered (Miller et al., 2013; Studer et al., 2015), the generation of induced neurons directly from fibroblasts more faithfully maintains epigenetic markers associated with the aging state (Mertens et al., 2016). Third, although neuronal induction bypasses key neurodevelopmental processes, perhaps failing to capture critical biology relevant for disease pathology, induced neurons have now been successfully applied to query neuronal deficits in autism (Chanda et al., 2013; Pak et al., 2015; Yi et al., 2016), bipolar disorder (Bavamian et al., 2015), Alzheimer's disease (Hu et al., 2015).

### Astrocytes

Once regarded as a population of cells providing little more than structural support to neuronal networks, the known roles of astrocytes in regulating neuronal function in the CNS is growing. It is now well-recognized that perturbed astrocyte function can exacerbate, and even cause, CNS diseases (Chung et al., 2015); for example, neuroinflammation and ischemia induce two different types of reactive astrocytes, termed A1 (“harmful”) and A2 (“helpful”) (Liddelow and Barres, 2017; Liddelow et al., 2017). Astrocytes are the most abundant cell type in the CNS and perform a wide variety of functions, including axonal guidance, response to inflammation, wound healing, and the formation of the blood brain barrier (Barres, 2008; Zhang and Barres, 2010; Verkhratsky et al., 2012; Freeman and Rowitch, 2013). Importantly, astrocytes are involved in recycling of glutamate and molecular regulation of ion, neurotransmitter and neurohormone concentrations, as well as synaptic pruning and maturity, underscoring their vital role in neuronal communication (Newman, 1995; Danbolt, 2001; Pfrieger, 2009). Astrocytes seem to function in an ordered manner to cover independent territory, contacting thousands of synapses through their multiple processes and branches (Bushong et al., 2002). In addition, these processes can be used to create connections with local capillaries and develop independent neurovascular units in which astrocytes mediate changes in CNS blood flow in response to neuronal activity (Schummers et al., 2008; Wolf and Kirchhoff, 2008; Koehler et al., 2009). Mirroring the diversity of their function, astrocytes display extraordinary heterogeneity in morphology, physiology, gene expression and developmental origin (Zhang and Barres, 2010). The number and complexity of astrocytes increase significantly with neuronal complexity in higher vertebrates, with important differences between rodents and humans that underscore the importance for cell-based systems to understand the contribution of this important cell type in disease pathology (Zhang et al., 2016). The astrocyte-to-neuron ratio increases with evolutionary complexity from low vertebrates to rodents and primates, with humans having a 46% higher glia-neuron ratio even when compared to other primates (Sherwood et al., 2006). Compared to their rodent counterparts, human cortical astrocytes are larger, display greater heterogeneity and diversity, and exhibit marked differences in their electrophysiological properties (Oberheim et al., 2009). When transplanted into mice, human glia (relative to transplanted mouse glia) improved learning and activity-dependent plasticity compared to controls (Han et al., 2013). These results underscore large and important differences between human and murine astrocytes, emphasizing the importance of developing clinically relevant, human-specific *in vitro* models that can help uncover important roles of human astrocytes in health and disease.

Strategies for the directed differentiation of astrocytes from hiPSCs can either rely on an NPC (Haidet-Phillips et al., 2011; Krencik and Zhang, 2011; McGivern et al., 2013; Serio et al., 2013; Shaltouki et al., 2013) or oligodendrocyte progenitor cell (Jiang et al., 2013) intermediate. Transplanted hiPSC-derived astrocytes integrate and function in the mouse brain *in vivo* (Haidet-Phillips et al., 2011; Krencik and Zhang, 2011; Jiang et al., 2013; Chen et al., 2015). Consistent with the late emergence of astrocytes during corticogenesis (Tabata, 2015), existing methods tend to be slow (up to 6 months), thus limiting their use for *in vitro* modeling (Krencik and Zhang, 2011; Jiang et al., 2013; Shaltouki et al., 2013). Induction of astrocytes from mouse fibroblasts occurs more rapidly (within 16 days), via overexpression of the transcription factors *NF1A, NF1B*, and *Sox9* (Caiazzo et al., 2015), the utility of this protocol in generating functional human astrocytes has not been demonstrated. hiPSCs have been differentiated to functional astrocytes for cell-based models of neuropsychiatric disorders *in vitro* (McGivern et al., 2013; Roybon et al., 2013; Serio et al., 2013; Shaltouki et al., 2013). Resulting hiPSC-derived astrocytes express canonical markers, participate in glucose homeostasis (Shaltouki et al., 2013), and can be engrafted into mouse striatum and upregulate expression of classical reactive astrocytic chemokines when treated with TNFα (Roybon et al., 2013). McGivern et al differentiated hiPSCs from patients with spinal muscular atrophy (SMA) into astrocytes, demonstrating that mutated SMA astrocytes had enlarged cell bodies with shorter processes and more pronounced GFAP expression, indicative of a reactive astrocyte phenotype (McGivern et al., 2013). In a similar fashion, Serio et al created astrocytes from hiPSCs carrying the TDP-43 mutation associated with ALS, and found that the resulting astrocytes had decreased survival, increased levels of TDP-43, and intracellular mislocalization of TDP-43 (Serio et al., 2013). Together, these studies demonstrate the utility of human and disease specific models for probing changes in astrocytic phenotype due to genetic or environmental challenges *in vitro*.

### Oligodendrocytes

Oligodendrocytes wrap neuronal axons in a thick membrane of myelin, enabling rapid conductance of electrical signals through neural networks. Myelination occurs as multi-step process that begins with proliferation and migration of oligodendrocyte precursor cells across large distances to the appropriate axon, synthesis of the myelin sheath itself, and finally wrapping and compaction of the insulating layer around the axon (Barateiro et al., 2016). The final steps of wrapping and compaction occur in the last weeks of gestation and the first postnatal months, with the bulk of the white matter in the CNS being produced during the first year of birth (Barateiro et al., 2016). Interestingly, myelinating capacity appears to be restricted to shortly after oligodendrocyte differentiation is completed (Watkins et al., 2008), such that the appearance of white matter in specific neuroanatomic regions appears to accompany maturation of cognitive function of that area (Nagy et al., 2004; Mabbott et al., 2006; Fields, 2008). Maintenance of myelin occurs throughout life, owing to the proliferation and activation of adult oligodendrocyte precursor cells (Zhu et al., 2007; Young et al., 2013); the inability to repair damaged myelin is implicated in myelin disorders such as ALS and multiple sclerosis (MS) (Barateiro et al., 2016). Oligodendrocytes communicate in a variety of ways with other cell types in the CNS; trophic factors secreted by oligodendrocytes modulate neuron axonal size and regulate distribution of ion channels in the axon (Barres, 2008), whereas gap junctions between astrocytes and oligodendrocytes allow exchange of small molecules that affect electrical coupling, and it appears that astrocytes play a role in the initiation of myelination (Orthmann-Murphy et al., 2008).

Although human oligodendrocytes can be generated from hiPSCs (Goldman and Kuypers, 2015) and are seemingly highly active once transplanted *in vivo* (Windrem et al., 2014), they myelinate less than 3% of axons *in vitro* (Kerman et al., 2015). Oligodendrocytes can also be generated through direct conversion methods in mice, via the overexpression of *Sox10*, oligodendrocyte transcription factor 2 (*Olig2*) and either *Nkx6.2* (Najm et al., 2013) or zinc-finger protein 536 (ZPF536) (Yang et al., 2013); the first method yielded oligodendrocyte restricted induced oligodendrocyte precursor cells, the second retained competence to generate astrocytes as well. It remains uncertain to what extent these same factors will be sufficient to generate induced oligodendrocyte precursor cells from human fibroblasts. Moving forward, methods to regionally pattern the fate and function of differentiated or induced oligodendrocyte precursor cells will be necessary.

### Microglia

Microglia maintain homeostasis throughout the CNS through active surveillance following by phagocytic clearance of debris and elimination of synapses during development in a process known as “synaptic pruning”; they also perform vital immune functions as the first line of defense in the nervous system. Using their extensive processes, microglia sweep the CNS parenchyma in search of unhealthy or diseased astrocytes and neurons that fail to express particular receptors and/or secrete “healthy” signals (Davalos et al., 2005; Sieger et al., 2012; Aguzzi et al., 2013; Prinz and Priller, 2014). Microglia measure neuronal activity using neurotransmitter receptors and immune activity by expressing receptors for chemokines and complement factors (Hanisch and Kettenmann, 2007); they respond to stimuli by altering migration, inflammatory response, cytokine release and phagocytic activity (Prinz and Priller, 2014). Microglia are the primary antigen presenting cells of the CNS, presenting antigens to infiltrating T lymphocytes through major histocompatibility complex (MHC) class II complexes (Butovsky et al., 2005). Activation of microglia in response to injury or disease results in a morphological change that differs depending on the signals detected and microglial modulators present (Hanisch and Kettenmann, 2007). Following tissue injury or in response to pathogens, non-neural macrophages undergo polarization into either (pro-inflammatory) or M2 (anti-inflammatory) macrophages. Likewise, activated microglia become polarized into M1-like and M2-like phenotypes; co-culture with M1 microglia leads to a cytotoxic phenotype in neurons and oligodendrocytes, while co-culture with M2 microglia promotes neurite outgrowth cells (Kigerl et al., 2009; Hu et al., 2012). However, microglia have difficulty undergoing polarization to an M2-like phenotype *in vitro* when compared to non-neural macrophages, hampering a complete understanding of these two different phenotypes (Kim et al., 2004; Durafourt et al., 2012). Interestingly, differences between M1 and M2 macrophages and microglia become difficult to appreciate in inflammatory and neurodegenerative diseases, with microglia co-expressing markers of both subtypes (Dal Bianco et al., 2008; Vogel et al., 2013).

Until recently, no directed differentiation protocols for the creation of microglia existed, limiting our understanding of this cell type to those studies performed using mouse models or post-mortem human tissue. Because microglia are derived from the primitive macrophages in the yolk sac of myeloid lineage (Alliot et al., 1999), they cannot be generated using neural progenitor cells (NPCs) as in other lineages discussed previously. Three recent reports have now detailed the creation of microglia from hiPSCs via a hematopoietic progenitor-like intermediate cell (Muffat et al., 2016; Abud et al., 2017; Pandya et al., 2017). While the protocols differed in their reliance on embryoid body differentiation and/or FACS, all three methods yielded immature microglia-like cells expressing canonical microglial markers and demonstrating phagocytic and migratory functionality (Muffat et al., 2016; Abud et al., 2017; Pandya et al., 2017).

### Summary

With a newfound ability to generate all of the major cell types of the CNS - neurons, astrocytes, oligodendrocytes and microglia—human hiPSC-based models are now primed to explore how the interactions of these various cell types contribute to risk for a variety of neuropsychiatric disorders. While post-mortem, animal models and hiPSCs have identified a number of cell autonomous deficits that underlie neurodegenerative (Marchetto et al., 2011; Bahmad et al., 2017; Poon et al., 2017) or psychiatric (Brennand and Gage, 2012; Ho et al., 2015; Wen, 2017) disorders, complex interactions between neural cells can now be explored in a fully human and patient-derived platform (Table 1). Below, we discuss evidence for non-cell autonomous effects in neurodegenerative disease (represented by ALS), psychiatric disease (represented by schizophrenia) and neurodevelopmental disease (represented by Rett Syndrome).

**Table 1:** Contribution of non-neuron cell types in neurodegenerative and neuropsychiatric diseases.

**Cell type**	**Physiological function**	**Representative role in disease pathology**	**Protocols**	**Challenges**
Astrocyte	Blood-brain barrier maintenance, glutamate, homeostasis, synaptic pruning, neurotransmitter reuptake, wound healing and response to inflammation.	ALS: Expression of mSOD1 in neurons is not sufficient to cause degeneration in mouse models of ALS, yet Selective deletion of mSOD1 from astrocytes confers slower disease progression in mice. Human wild type neurons undergo degeneration *in vitro* when co-cultured by mSOD1 in astrocytes. Schizophrenia: Loss of astrocytes associated with schizophrenia as are altered gene sets involved in astrocyte function. Rett syndrome: MeCP2 deficiency in astrocytes causes abnormalities in neural cytokine production impacting development. hiPSC derived astrocytes derived from patients with Rett syndrome have deficiencies in microtubule transport that are abrogated by drugs shown to have behavior correcting effects in mice.	(1) Directed differentiation from hiPSCs through NPC intermediate (Haidet-Phillips et al., 2011; Krencik and Zhang, 2011; McGivern et al., 2013; Serio et al., 2013; Shaltouki et al., 2013). (2) Directed differentiation from hiPSCs through oligodedrocyte progenitor (Jiang et al., 2013). (3) Transcription factors induction of fibroblasts in mice (Caiazzo et al., 2015).	Slow culture/differentiation time for hiPSC based protocols (~6 months). No fibroblasts-induction protocols available for human cells. Poorly understood role of astrocyte heterogeneity in disease limits ability to recapitulate these effects *in vitro*.
Oligodendrocyte	Myelination of neuronal axons in the CNS to enable conductance of electrical signals. Proliferation and activation of oligodendrocyte precursor cells (OPC) occurs throughout life.	Schizophrenia: Changes in oligodendrocyte density, differentiation and morphology in post-mortem tissue studies. Rodent *in vitro* studies show genes associated with Schizophrenia (e.g., DISC1 impact oligodendrocyte differentiation.	(1) Directed differentiation from hiPSCs (Goldman and Kuypers, 2015). (2) Direct conversion by overexpression of Sox10, Oligo2 together with either Nkx6.2 or ZPF536 in mice (Najm et al., 2013; Yang et al., 2013).	No available protocols for the conversion of oligodendrocyte from fibroblasts in humans. Myelination may be restricted to period immediately following OPC maturation.
Microglia	Perform synaptic pruning and play critical immune role by removing diseased neurons and glia serving as antigen presenting cell of CNS. Change phagocytic activity and mediate inflammatory reaction in response to neurotransmitter signals in CNS.	ALS: Selective removal of mSOD1 from microglia extends lifespan in mouse model of ALS. Rett Syndrome: murine Rett syndrome microglia have neurotoxic effects in co-cultured with hippocampal neurons. hiPSC derived microglia carrying MECP2 deletion are smaller potentially limiting their ability to perform critical immune surveillance functions.	(1) Directed differentiation via hematopoietic progenitor (Muffat et al., 2016; Abud et al., 2017; Pandya et al., 2017).	Little known about *in vitro* microglia phenotypes due to historical difficulty culturing human primary cells.

*Representative examples from both in vitro and in vivo studies in mice and human are included, as well as relevant citations for protocols to generate these cell types through directed differentiation of hiPSCs or through reprogramming of other cell types*.

## Non-cell autonomous effects in neuropsychiatric disorders

### Amyotrophic lateral sclerosis (ALS)

ALS is a debilitating neurodegenerative condition causing muscle atrophy and loss of control of motor function, rendering patients paralyzed and eventually unable to breathe (Brooks, 1996; Appel et al., 2011). The symptomatic phase of ALS is associated with massive activation of microglia and astrocytes, which destroy motor neurons of the CNS (Kushner et al., 1991; Nagy et al., 1994; Schiffer et al., 1996). Though there is undoubtedly a genetic component to the disease, the majority of cases are idiopathic and with no family history, although a handful of highly penetrant monogenic forms of ALS follow Mendelian inheritance patterns (Brown, 1997; Cole and Siddique, 1999). The initial signs of ALS are mild, and so many patients are not identified until the damage to the tissue is quite significant. Altogether, the complex genetics, unclear environmental compounds and late diagnoses of ALS patients have made understanding the onset of disease pathology very difficult to date.

Mutations in the Super Oxide Dismutase gene (*SOD1)* lead to a dominant, inherited form of ALS (Rosen et al., 1993), and are well-studied with the hope that by focusing on the most penetrant and significant genetic defects associated with ALS, we may come to understand broader aspects of ALS disease onset and progression that are relevant to other ALS-associated mutations. Initial experiments in rats with mutant *SOD1* gene (*mSOD1*) displayed degenerative symptoms and pathology consistent with ALS (Nagai et al., 2007). Moreover, introduction of the mutant gene into motor neurons in the CNS did not cause neurodegeneration when motor neurons were surrounded by healthy support cells, but even those wild type neurons proximal to mutant glia underwent degeneration (Clement et al., 2003). These data suggest that ALS disease onset and progression might be mediated by negative interactions between various cell types of the CNS, rather than an intrinsic dysfunction in neurons themselves. In fact, selective deletion of the m*SOD1* gene from mouse astrocytes in transgenic mice conferred a slower disease progression (Yamanaka et al., 2008). More recently, microglia have also been identified as key mediators of ALS progression; selective removal of the mSOD1 gene from microglia significantly extended lifespan (Boillée et al., 2006). Interestingly, while the interaction between neurons and microglia appears to be protective at first, cellular stress resulting from misfolded mSOD1 ultimately activates microglia to a proinflammatory and neurotoxic phenotype (Appel et al., 2011).

Consistent findings were subsequently observed using *in vitro* models of ALS. Mouse astrocytes expressing the mSOD1 gene have a toxic effect on wild type neurons *in vitro*; this effect was stronger in motor neurons than neuronal cell types (Di Giorgio et al., 2007; Nagai et al., 2007). Expression of mSOD1 in neurons alone is insufficient for neurodegeneration. To observe significant injury, mSOD1 must be expressed in glia; moreover, the presence of wild type non-neuronal cells delays degeneration (Clement et al., 2003; Zhao et al., 2010). Sandwich co-cultures of mouse embryonic stem cell (ESC)-derived motor neurons and primary glia allows controlled interaction and subsequent separation of these two cell types, identifying striking differences in autonomous and non-autonomous changes in gene expression (Phatnani et al., 2013).

Ultimately, many of these results have now been reproduced in a human context. As in mouse ESC-derived motor neurons, human ESC-derived motor neurons are also selectively more sensitive to toxic non-cell autonomous effects than interneurons (Di Giorgio et al., 2008). This toxicity was correlated with changes in glial gene expression, which was used to identify candidate molecules associated with the toxic mutant glia-associated effects. For example, when prostaglandin D2 was added exogenously to motor neurons co-cultured with unaffected glia there was a reduction in survival of motor neurons; however, when administered in the presence of a prostaglandin D2 inhibitors, those co-cultures containing mutant astrocytes had better survival relative to control. Similar experiments in which ESC-derived motor neurons were co-cultured with primary mSOD1-expressing human glia also showed a specific decrease in motor neurons, with no adverse effects on other neural cell types (Marchetto et al., 2008). The toxicity of mSOD1-expressing astrocytes was due, at least in part, to reactive oxygen species (ROS) generated by the glia; overexpression of *NOX2* increased oxygen radicals, an effect could be reversed using apocynin, a NOX2 inhibitor, preventing the loss of motor neurons when co-cultured with mSOD1-expressing glia.

Although the m*SOD1* gene has now been conclusively linked to aberrant glia function and subsequent motor neuron death in ALS, it remains unclear to what effect the other familial and sporadic forms of ALS arise due to astrocyte malfunction. The ongoing generation of a library of ALS patient-specific hiPSCs should provide a powerful tool to interrogate how diverse mutations identified in ALS patients influence glial/neuron interplay within the CNS, and how this contributes to disease pathology. A growing number of recent studies have reported significant changes in mitochondrial function, synapse organization, receptor binding, and neuronal health in ALS-patient derived motor neurons (Dimos et al., 2008; Egawa et al., 2012; Chestkov et al., 2014; Alves et al., 2015). Recently, Meyer et al. reported a method to induce fibroblasts from ALS patients with two independent diseases-associated mutations into astrocytes; relative to controls, these induced astrocytes showed increased toxicity in co-culture with mouse motor neurons *in vitro* (Meyer et al., 2014). Yet the story of astrocyte/neuron interplay in ALS may be mutation-specific, as co-culture of hiPSC-derived astrocytes and motor neurons carrying a separate ALS causing mutation, TDP-43, revealed that mutant TDP-43 astrocytes did not cause adverse effects on neuronal survival. These results highlight the importance of modeling a variety of ALS-associated mutations, in order to understand the full complexity of processes contributing to neurodegeneration. As gene editing and hiPSC technologies improve, it will become increasingly feasible and important to conduct larger high throughput experiments, in order to understand how various ALS causing mutations affect neuronal health and function in a human disease context.

### Schizophrenia

Schizophrenia is a debilitating neuropsychiatric disorder present in 1% of the world population and is associated with increased risk of homelessness, unemployment and suicide (Kooyman et al., 2007; Foster et al., 2012). Symptoms typically present in the early stages of adult life and include but are not limited to psychosis, reduced social engagement and lack of motivation that present a high personal cost and risk to the patient (Lewis and Lieberman, 2000). Post-mortem studies of neural tissue, as well as MRI studies on patients with schizophrenia reveal reduced brain volume, spine density and abnormal neural distribution and connectivity, particularly in the prefrontal cortex and hippocampus (Benes et al., 1991; McCarley et al., 1999; Lewis and Lieberman, 2000; Hulshoff Pol et al., 2002). Both the molecular mechanisms and cell types involved in schizophrenia pathology are complex, confounding the development of novel pharmacological treatments. While the high heritability of schizophrenia (80–85%) points to a strong genetic component, the disorder is highly polygenic and associated with rare highly penetrant mutations as well as common variations of smaller effect; schizophrenia is significantly associated with at least 108 different genetic loci (Schizophrenia Working Group of the Psychiatric Genomics Consortium, 2014) of various effect sizes. The genetic complexity of this disorder necessarily limits the development and applicability of mouse models for this psychiatric disorder, making necessary the creation of human cell-based scalable platforms capable of recapitulating critical aspects of disease initiation and progression.

The diverse neural regions implicated in its pathology suggest that symptoms may be indicative of underlying problems with neural communication involving various cell types. hiPSC-based studies of schizophrenia to date have generally focused on cell-autonomous deficiencies in neuron differentiation (Robicsek et al., 2013; Yoon et al., 2014; Brennand et al., 2015) maturation (Brennand et al., 2011) and function (Wen et al., 2014; Yu et al., 2014). Nonetheless, a growing role for aberrant astrocyte function (Matute et al., 2005), microglia activity (van Berckel et al., 2008) and decreased myelination (Bernstein et al., 2015) is now being appreciated in clinical studies, although it is unclear whether these reflect a cause or consequence of symptom onset. Our hope is that hiPSC-based technologies will provide a tractable, modular platform to establish causal links between schizophrenia genetic predisposition, cell-type interactions and disease-relevant phenotypes.

Magnetic resonance imaging (MRI) and non-volumetric diffusion tensor imaging (DTI) of post-mortem tissue have revealed abnormalities in white matter on a macro and microstructure level respectively (Walterfang et al., 2011; Whitford et al., 2011), prompting an increased interest in the role that oligodendrocytes may play in schizophrenia. Numerous studies have now associated schizophrenia with decreased oligodendrocyte differentiation (Mauney et al., 2015) and density (as much as 30% reduced in some brain regions) (Vikhreva et al., 2016), as well as changes in oligodendrocyte morphology and spatial distribution in patient brains (Uranova et al., 2001; Schmitt et al., 2009). Moreover, *in vitro* rodent studies demonstrated that the schizophrenia-associated gene DISC1 impacts oligodendrocyte differentiation (Hattori et al., 2014) and human genetics have identified oligodendrocyte gene sets associated with schizophrenia, most of them related to fatty acid and cholesterol metabolism (Goudriaan et al., 2014). To date, no hiPSC- or ESC-based technologies have examined the effect of schizophrenia-associated variants on oligodendrocyte differentiation and function, which could shed light on how the differences in white matter organization and myelination observed in post-mortem tissue arise on the cellular level.

Astrocytes are believed to contribute primarily to neuroinflammation and synaptic maturation/pruning in the human brain (Freeman and Rowitch, 2013), processes that are increasingly linked to schizophrenia (Jaaro-Peled et al., 2010; Fineberg and Ellman, 2013). Astrocyte loss has also been associated with schizophrenia in various cortical and subcortical regions of the brain, particularly in the white matter (Rajkowska et al., 2002; Williams et al., 2013a,b). Along with the gene sets involved in oligodendrocyte function, additional gene sets involving astrocyte function are altered in schizophrenia (Goudriaan et al., 2014). Post-mortem pathology inevitably leads to tissue shrinkage (“fixation artifacts”), particularly in regions that have abundant astrocyte cell processes (Garman, 2011); despite this technical limitation, a few post-mortem studies have observed subtle reductions in glial cell volume (Rajkowska et al., 2002) and cell number (Stark et al., 2004) in schizophrenia cortical brain tissue, although these results are not yet widely accepted. Postmortem cortical expression (Barley et al., 2009) and protein levels (Steffek et al., 2008) of major astrocyte associated genes such as glial fibrillary acidic protein (*GFAP*) are perturbed in schizophrenia. Critically, the relevance of these post-mortem observations is clouded by the fact that antipsychotic treatments may impact astrocyte levels and/or function in the CNS, as these cells express dopamine receptors (Hertz et al., 1984).

The use of hiPSC-based models for schizophrenia continues to be increasingly useful; we and others have demonstrated increases in oxidative stress, (Paulsen et al., 2012; Robicsek et al., 2013; Brennand et al., 2014) deficits in adherens junctions and polarity (Yoon et al., 2014), as well as marked differences in migration and responses to environmental stressors in NPCs derived from schizophrenic patients (Brennand et al., 2014; Hashimoto-Torii et al., 2014). Similarly, schizophrenia hiPSC neurons exhibit decreased synaptic maturation and neurite number (Brennand et al., 2011; Robicsek et al., 2013; Wen et al., 2014; Yu et al., 2014), as well as a reduction in synaptic activity (Wen et al., 2014; Yu et al., 2014). Given the success of these previous hiPSC models and the strong evidence presented above on the role of glial cell-types in schizophrenia disease pathology, it may be very informative to extend these *in vitro* systems to query glial function using patient-specific hiPSC lines to develop better, more human-specific models of schizophrenia.

### Rett syndrome

Rett Syndrome is a rare X-linked neurodevelopmental disorder affecting the gray matter of the brain, primarily in female patients, and characterized by an initial period of normal development during early infancy followed by a sudden attenuation of developmental growth and the loss a number of motor and language skills (Hagberg et al., 2002). This regression of physical development is associated with differences in cognitive function; many patients exhibit autistic-like behaviors, sleep disorders and increased anxiety (Hagberg et al., 2002; Bienvenu and Chelly, 2006). Rett Syndrome is frequently caused by *de novo* mutations in the methyl CpG binding protein 2 gene (*MECP2*), though other genetic variations account for a minority of cases. *MECP2* encodes the regulatory protein MeCP2, which binds to methylated DNA to regulate transcription of a number of genes (Amir et al., 1999; Bienvenu and Chelly, 2006). How such broad changes in transcriptional regulation leads to the neurodevelopmental and neurobehavioral phenotypes of Rett syndrome is not fully clear, but the unigenic nature of the disorder makes it particularly tractable to study using hiPSC-based technologies.

The role of *MECP2* mutations in neurons has been well-examined in a variety of studies (Marchetto et al., 2010; Kim et al., 2011; Farra et al., 2012), but mutant *MECP2* also exerts its effect through non-cell autonomous events involving astrocytes. MeCP2 deficiency in astrocytes causes significant abnormalities in cytokine production and neuronal dendritic induction that could impact neurodevelopment in mice (Maezawa et al., 2009). Mutant astrocytes have an adverse effect when co-cultured with either wild type or mutant hippocampal neurons, an effect that can be recapitulated using conditioned media alone (Ballas et al., 2009), suggesting that astrocyte targets of MeCP2 regulation are involved in glial maintenance of neuronal function and that astrocytes are a key player in Rett syndrome pathology. Importantly re-expression of *MECP2* preferentially in astrocytes restores normal neuronal dendritic morphology, improves locomotion and anxiety, and rescues respiratory symptoms in a mouse model of Rett syndrome (Lioy et al., 2011).

While the role of astrocytes in non-cell autonomous effects on neuronal function in Rett syndrome is well-established in mice, it was unclear whether the same effects held true in a human context. Two recent studies using Rett syndrome patient-derived hiPSCs have now not only confirmed that astrocyte role in Rett Syndrome, but also identified molecular targets involved in astrocyte-mediated neuronal deficits. Astrocytes derived from patient-specific hiPSCs recapitulate the negative effects on neuronal morphology observed in mouse studies, an effect that can again be reproduced using only conditioned media (Williams et al., 2014). Using combinations of control and Rett Syndrome-derived astrocytes and interneurons, the authors show that the glial effect on neuronal function is independent of intrinsic deficits in neurons themselves and demonstrated that insulin-like growth factor (IGF-1) and GPE (a short peptide containing the first 3 amino acids of IGF-1) are capable of partially rescuing the astrocyte-mediated neuronal phenotypes. The power of hiPSC-based systems to probe non-cell autonomous systems in Rett syndrome is underscored by recent studies demonstrating that MECP2-mutant astrocytes from patient-derived hiPSCs have deficiencies in microtubule-dependent vesicle transport, and that administration of Epothilone D, a microtubule stabilizing agent capable of crossing the blood brain barrier is sufficient to restore microtubule dynamics in these cells (Delépine et al., 2016). Critically, weekly doses of Epothilone D was capable of reversing the reduced exploratory behavior in a mouse model of Rett Syndrome (Delépine et al., 2016), demonstrating the power of hiPSC-based platforms to identify new targets that are capable of improving animal behavior when administered *in vivo*.

Microglia have also been implicated in Rett syndrome pathology through a variety of *in vitro* and *in vivo* mouse models. Selective correction of Rett syndrome microglia with wild type microglia using a Cre-Lox based system in mice produced an improvement of symptoms, one that could be reversed using annexin IV, suggesting that microglia-mediated phagocytosis is a key mechanism of Rett syndrome pathology (Derecki et al., 2012). In addition, murine Rett syndrome microglia appear to have a direct neurotoxic effect on hippocampal neurons in co-culture models, releasing increased glutamate that causes stunted dendritic morphology and disruption of microtubule organization that ultimately disrupts synaptic function (Maezawa and Jin, 2010). While these findings have not yet been tested in a human context, it was recently shown that hiPSC-derived microglia carrying the *MECP2* deletion are significantly smaller than their wild type counterparts (Muffat et al., 2016), which may impart a decreased ability to “patrol” the parenchyma and clear apoptotic tissue. Further studies using this newly developed pathology may offer important insight into Rett syndrome pathology and provide a powerful platform to identify molecular targets for drug development.

Altogether, while Rett syndrome is arguably the first autism spectrum disorder to be convincingly linked to aberrant astrocyte and microglial function, there is no reason to suspect that other forms of autism will not soon be associated with neuron non-cell autonomous effects as well. Complex genetic neuropsychiatric disorders, from ALS to schizophrenia to Rett Syndrome, may in fact represent a convergence of clinical phenotypes arising through a diverse range of genetic and cellular mechanism, suggesting that a “personalized” or genotype-dependent understanding of disease mechanisms may be critical for properly matching patients with appropriate therapeutics.

## The future of hiPSC-based drug screening

The development of new pharmaceutical treatments for neuropsychiatric diseases has been severely hampered by the poor availability of preclinical models that adequately capture the complex pathophysiology of these disorders. It has been difficult to translate the success of the high throughput drug screens used to identify novel targets and lead structures for therapeutics in other fields to neuroscience. Moving forward, we hope that this will be addressed by this new ability to create disease specific, patient-derived hiPSC lines, which serve as genetically relevant models that are scalable and easily perturbed through genetic and chemical approaches. Importantly, the ability to create and bank large stores of hiPSC-derived cells will permit repeated experiments across genetically isogenic human cell types, hopefully improving reproducibility. As advancements in high content imaging technologies develop alongside hiPSC technology, this integrated approach may help identify new therapeutic targets for drug development and advance our understanding of genotype-phenotype correlations in neuropsychiatric disease in ways that were not previously possible. This approach is of particular importance for complex genetic diseases such as schizophrenia and autism, where simplistic knock in models fail to accurately capture each patient's complex and potentially unique genetic background.

Pluripotent stem cell based screens have shown notable recent successes in the context of neuropsychiatric disease, reviewed elsewhere (Haggarty and Perlis, 2014; Haggarty et al., 2016). In the context of mitochondrial DNA (mtDNA) disorders, NPCs derived from patients carrying a single base pair mutation in the MT-ATP6 gene have defective ATP production, elevated mitochondrial membrane potential, and dysregulation of calcium handling (Lorenz et al., 2017); a large high-throughput screen using FDA approved drugs identified avanafil as a potential therapeutic owing to its ability to restore normal calcium homeostasis. The introduction of a TCF/LEF-responsive luciferase reporter into hiPSC-derived NPCs permitting screening of a small pilot library of 1500 compounds, identifying a number of compounds that potentiate Wnt or lithium signaling (Zhao et al., 2012), with potential relevance to a number of psychiatric disorders (including schizophrenia and Fragile X) and many common antidepressants and antipsychotics. A similar approach, applying an ATP bioluminescence end-point assay to screen a 1,000 compound library, identified five novel compounds that enhance proliferation and viability of hiPSC-derived NPCs (McLaren et al., 2013); these compounds could be used to further expand populations of NPCs and facilitate larger screens moving forward.

While the screens outlined above are unique in their methods and targets of interest, they are unified in their focus on a single cell type, limiting their relevance to the physiological context. With advancements in hiPSC differentiation protocols now encompassing many different neural cell types, we hope that future screening experiments will combine several distinct pre-differentiated cell types. Such a proof-of concept screen was conducted using Nestin-positive progenitor cells that were differentiated into neurons and/or astrocytes, cultured both in isolation and together (Efthymiou et al., 2014); a high-throughput MTT assay measured viability, while live-cell imaging tracked organelles within neurons and examined neurite length as a proxy for neuronal maturation. Likewise, a large 2.4 million compound screen using mouse ESC derived NPCs differentiated into a mix of neuron and glial subtypes used a fluorimetric imaging plate reader to measure calcium influx and screen for potentiators of AMPA receptor signaling (McNeish et al., 2010); however, the analysis did not stratify hits based on cell type, potentially diluting more modest effects that may have had large effects within key cell types. Overall, given our growing understanding of non-cell autonomous effects on neuronal health, next generation screening platforms must address the challenge of stratifying by cell type and considering interactions between cell types within a single population of cells. Toward this, an elegant platform that allows for the targeting of safe harbor loci in hiPSCs to introduce multiplexed lineage-specific reporter systems on the same isogenic background (Pei et al., 2015) could be combined with high content imaging technologies, providing a valuable context to resolve which cell populations are affected by candidate molecules. The importance of this analysis is demonstrated in a follow-up paper by the same group, which showed that of an initial panel of 80 compounds, 50 were observed to have a cytotoxic effect in at least one cell type but only four of those showed cytotoxicity in four different neural subtypes (Pei et al., 2016). Given the disparate and cell-specific phenotypes discussed above in a variety of neuropsychiatric disorders, qualifying successful “hits” on high throughput screens by matching the drug target with the cell type will be of critical importance for pharmaceutical development.

**Figure 1:**
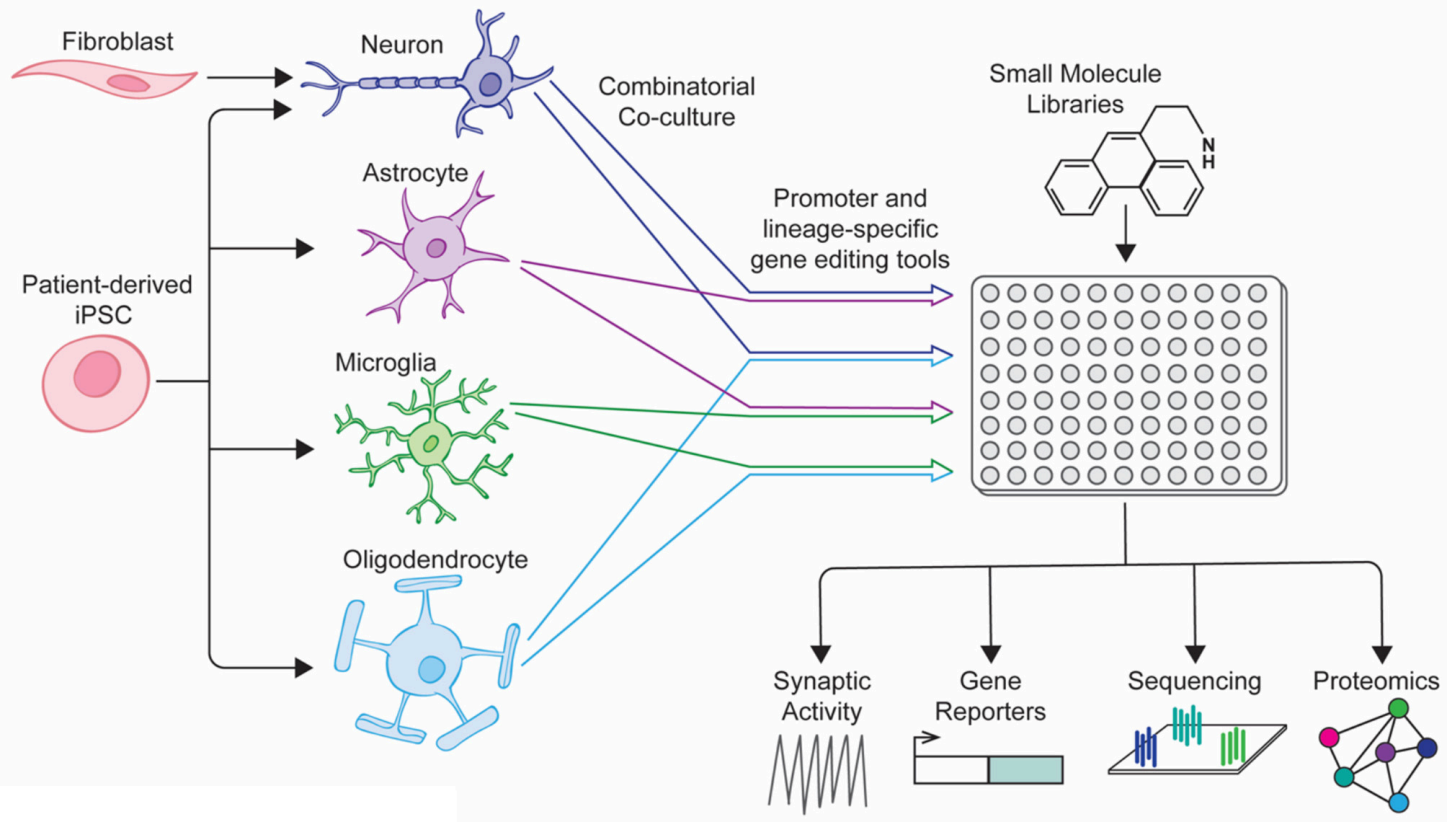
Next-generation drug screening platform for neuropsychiatric disorders. Directed differentiation and reprogramming of patient-specific cell types into various neural cell types allows for combination culture of cell types of interest. When combined with lineage-specific reporter systems, mixed neural cultures could be screened using small-molecule libraries and the results of downstream assays correlated with the cell-type of interest.

A number of coculture platforms have been developed for the study of neurological disease, shedding light on the interactions that occur between neural cell types (excellently reviewed by Meyer and Kaspar, 2017). These vary in their design and complexity, from focusing on paracrine signaling through transwell interactions to culturing disparate cell populations in pre-determined orientations to modeling physiological structures such as the blood brain barrier. Recently, a number of groups have reported protocols for the generation of endothelial cells, pericytes, neurons and astrocytes involved in the blood-brain barrier (BBB) from hiPSCs and other sources, using transwells to study efflux of a variety of drugs across the newly formed barrier (Lippmann et al., 2012; Boyer-Di Ponio et al., 2014; Appelt-Menzel et al., 2017; Yamamizu et al., 2017). When combined with microfluidic systems similar to those reported in Wang et al, these coculture systems may provide an efficient and cost-effective model to screen large pharmaceutical libraries (Wang et al., 2017). Newer, more sophisticated screening platforms should take inspiration from these co-culture models, adapting platforms to a high-throughput format that can be combined with fluorescent reporters and high content imaging software, allowing exploration of how the interactions between cell types change under the influence of pharmaceutical compounds (Figure 1). With ever improving cellular differentiation protocols allowing us to generate larger numbers of defined neural cell types with increasing efficiency, future screens should improve our understanding of neurological disease pathology and identify new treatment modalities for neurodegenerative and neuropsychiatric disease.

## Summary

New advances in the field of stem cell biology and reprogramming are allowing for the creation of once inaccessible neural cell types, improving our ability to model complex neuropsychiatric and neurodegenerative diseases in ways not previously possible. The development of these models has begun to shed light on important interactions between neural cell-types in the context of human disease pathology. As the importance of these non-cell autonomous effects becomes increasingly clear, drug discovery platforms that pair new stem cell technologies with high throughput assays and high content imaging software capable of analyzing individual cell populations within a heterogenous pool may be an invaluable resource for the development of new therapies for these complex disorders.

## Author contributions

DG and KB: wrote the manuscript; JG: prepared Figure 1.

### Conflict of interest statement

The authors declare that the research was conducted in the absence of any commercial or financial relationships that could be construed as a potential conflict of interest. The reviewer LJ and handling Editor declared their shared affiliation.
